# Immunomodulatory Effect of Traditional Chinese Medicine Combined with Systemic Therapy on Patients with Liver Cancer: A Systemic Review and Network Meta-analysis

**DOI:** 10.7150/jca.74829

**Published:** 2022-09-06

**Authors:** Qing Pu, Lihua Yu, Xinhui Wang, Huiwen Yan, Yuqing Xie, Yuyong Jiang, Zhiyun Yang

**Affiliations:** Center of Integrative Medicine, Beijing Ditan Hospital, Capital Medical University, Beijing, China

**Keywords:** Traditional Chinese Medicine, systemic therapy, immunoregulation, liver cancer, randomized controlled trials, network meta-analysis.

## Abstract

**Objective:** As immune combination therapy in the treatment of liver cancer made significant achievements, and the modulating effect of traditional Chinese medicine (TCM) on immunity gradually appeared. The main purpose of this study was to study the effect of different TCM combined with systemic therapy (ST) on immune regulation in patients with liver cancer, as well as the efficacy and safety of combined therapy, and to find the best combined application scheme by ranking.

**Methods:** Nine electronic databases were searched from January 1, 2010, to November 12, 2021, to search for RCTs of TCM combined ST in the field of liver cancer for literature screening, quality evaluation and data extraction. STATA 15.0 and RevMan 5.3 software were used to conduct network meta-analysis to analyze and explore the significance of TCM combined ST in immune regulation, efficacy and safety in clinical application. The probability value of the surface under the cumulative ranking curve was used to rank the processing studied.

**Results:** A total of 25 studies involving 2,152 participants were included in the network meta-analysis, including six traditional Chinese medicine injections and seven proprietary Chinese medicines. The results showed that Dahuang Zhechong Wan and Kangai injection combined with ST were the best choices for immune regulation. Moreover, the Huaier granule was the best choice to reduce vascular endothelial growth factors.

**Conclusion:** For patients with liver cancer, TCM combined with ST was better than that of ST alone and can significantly improve the immune function of patients as well as the efficacy and safety of treatment. However, given the limited sample size and methodological quality of the trials that we included in our study, more centralized and randomized controlled trials with a large sample size are required to verify our findings.

## Introduction

Liver cancer is one of the most common malignant tumors worldwide, with an insidious onset and a high degree of malignancy. Most patients are diagnosed in the middle and late stages of the disease, when the opportunity for surgical resection is lost 1. According to the guidelines 2, systemic therapy (ST) is the main treatment method for intermediate and advanced liver cancer. This method achieves the purpose of anti-tumor through systemic drug action, which is divided into first-line treatment and second-line treatment. The main types of drugs include immune checkpoint inhibitors, multi-kinase inhibitors and systemic chemotherapy drugs. Among them, "T + A" (Atezolizumab + bevacizumab) combined immunotherapy achieved the most significant efficacy, its effectiveness and safety are the best, is the first-line treatment. The anti-tumor effect can be achieved by blocking the PD-1/PD-L1 pathway to restore the killing effect of killer T cells in the tumor microenvironment and promote the initiation and activation of T cells and inhibit tumor angiogenesis. Compared with sorafenib alone, combined immunotherapy was more effective in prolongating survival time (Overall survival (OS): 19.2 vs 13.4 (month), Hazard ratio (HR): 0.66; Progression-free survival (PFS): 6.9 vs 4.3 (month), HR: 0.65) 3. Effective regulation of immunity has become an important way to treat liver cancer, and various combination therapies are also being actively explored.

**Figure 1:**
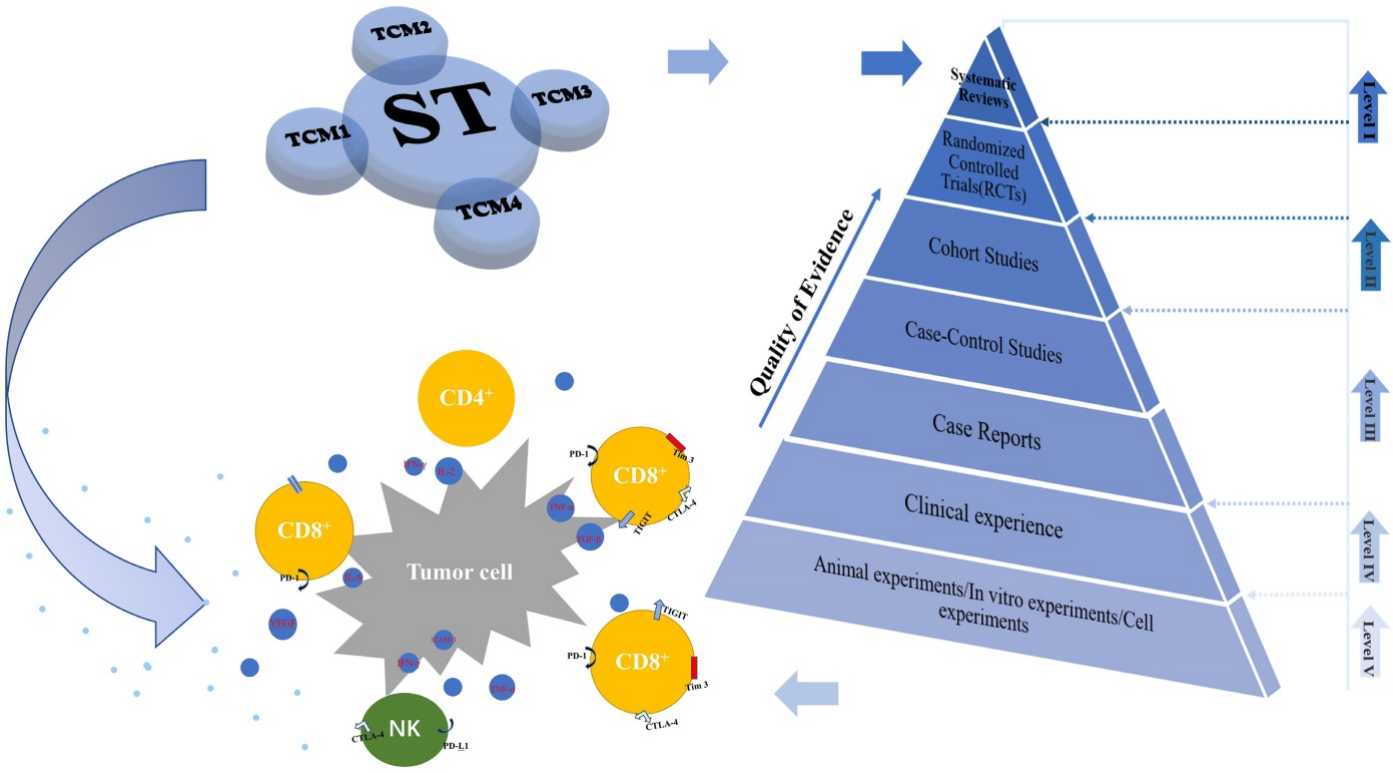
** Graphical abstract of the network meta-analysis.** Note: TCM, traditional Chinese medicine; ST: systemic therapy.

In China, traditional Chinese medicines (TCM) have been widely used in the treatment of cancer, which has important clinical significance in improving adverse reactions (ADRs), improving the survival rate and quality of life of patients, and inhibiting tumor growth 4, 5. A number of studies 6-8 have shown that TCM can inhibit the growth and metastasis of tumor cells and promote apoptosis of tumor cells by upregulating the immune response. One research showed that astragaloside, an active compound made from the traditional herbs astragalus, promotes the expression of CD25 and CD69 on CD4^+^T cells by increasing Interferon-gamma (IFN-γ) and T-box transcription factor (T-bet) mRNA levels in spleen cells and increasing Interleukin-2 (IL-2) and IFN-γ secretion, which subsequently increased T-cell activity and enhances immune function 9. Another research showed that the Wan-Nian-Qing prescription, a commonly used treatment for malignant tumors that consists of the compound traditional Chinese medicines, its main components can regulate serum levels of interleukin, chemokines and tumor necrosis factor, thereby activating natural killer cells (NK) and regulatory T cells, promoting tumor cell apoptosis, and ultimately inhibiting the growth of liver cancer 10. Studies have shown that Icariin, a generic flavonoid compound, inhibits the development and growth of liver cancer by regulating the IL-6/JAK2/STAT3 pathway 11 and also inhibits the expression of PD-L1 by targeting protein IκB kinase α, thus regulating immunity 12. There are many forms of TCM, including proprietary Chinese medicine, TCM injections (CHIs) and TCM decoction. TCM decoction is often added or subtracted according to different doctors' experience and medication habits, which leads to the unfixed composition and cannot be classified uniformly. In contrast, proprietary Chinese medicines and CHIs with fixed composition are more suitable for meta-analysis and clinical recommendation. In the treatment of liver cancer, TCM is often combined with conventional therapy, and TCM combination therapy has proved to be superior to conventional therapy alone 13, 14. Several meta-analyses have compared the therapeutic efficacy of proprietary Chinese medicines or CHIs combined with local therapy 15, 16. In view of the continuous development of liver cancer immunotherapy and the considerable prospect of TCM adjuvant ST to regulate immunity, moreover, there is no clinical trial to directly compare immunomodulatory effects of different TCM combined ST. In this study, a network meta-analysis (NMA) was designed and implemented to address this knowledge gap. Through this study, we aimed to determine the best TCM combined with ST to regulate immune strategy and provide a more powerful basis for future clinical practice. The graphical abstract of the NMA is shown in Figure 1.

## Materials and methods

The current NMA procedures were implemented strictly according to the Preferred Reporting Items for Systematic Evaluation and Meta-Analysis (PRISMA) guidelines 17. The complete PRISMA checklist is included in Supplementary Material document 1.

### Database and Retrieval Strategies

Randomized clinical trials (RCTs) of TCM combined with ST for liver cancer patients were determined through literature search. Nine databases (including five English databases and four Chinese databases) that were searched for the NMA in the study were as follows: PubMed, PubMed Central, the Cochrane Library, Embase, Web of Science, the China National Knowledge Infrastructure Database, the Wan-fang Database, the Cqvip Database, and the Chinese Biomedical Literature Database. The retrieval period ranged from January 1, 2010, to November 12, 2021. It should be noted that there is little relevant literature in the English database, so no time limit is set for retrieval. To determine the relevant literature and conduct a comprehensive search, we applied a combination of the Medical Subject Headings (MeSH) with free words, which focused on the following themes: “liver cancer”, “traditional Chinese medicine”, “systemic therapy”, and the names of the various types of drugs they're associated with them, including proprietary Chinese medicine, CHIs, immune checkpoint inhibitors and multi-kinase inhibitors, etc... Take the PubMed search process as an example, the specific search terms are shown in Supplementary Material Document 2.

### Selection Criteria

Eligible studies were accurately identified according to the PICOS (patients, intervention, comparison, outcome, and study design) format of PRISMA guidelines. 1) All patients included in this study were histopathologically diagnosed with liver cancer, regardless of sex, age, race, region, or nationality; 2) The control group received only ST, including immunotherapy, targeted therapy, and systematic chemotherapy, while the experimental group received CHIs or proprietary Chinese medicines on the basis of the control group; 3) Study outcomes at least include immune-related indicators, which are the main outcomes; 4) The study type was RCT. The exclusion criteria were as follows: 1) Non-liver cancer patients, patients with other tumors, or patients with other serious systemic diseases; 2) The research contents (including pharmacological mechanism studies, animal experiments and cell experiments, etc.) and interventions (including surgical treatment, local radiotherapy, and hepatic arterial chemoembolization) were unrelated; 3) The study design and publication type were non-RCT (including case control studies and case reports), reviews, meeting abstracts); 4) Outcome indicators were not relevant or the data were incomplete. and 5) Full text unavailable or duplicated.

### Types of Outcomes

The main outcomes were immune-related indicators, including T-lymphocyte subsets (CD3^+^, CD4^+^, CD8^+^, CD3^+^/CD8^+^, and CD4^+^/CD8^+^), NK, and cytokines (vascular endothelial growth factors [VEGF] and other inflammatory factors). Secondary outcomes included the clinical effectiveness rate, Quality of Life, ADRs, tumor markers (alpha fetoprotein [AFP]), and half-year and one year survival rates. The statistical methods of the outcomes indicators were as follows: 1) Immune-related indices and tumor markers that were calculated from the change in value before and after treatment; 2) the clinical effectiveness rate and Quality of Life. According to the Objective Response Criteria in Solid Tumors by WHO, the clinical effectiveness rate = [number of complete response patients + partial response patients] / total number of patients × 100%. In accordance with the KPS functional status scoring criteria, an increase of more than 10 points in the KPS score was considered as a significant improvement in Quality of Life; 3) ADRs and survival rate = events / total × 100%.

### Data Extraction and Quality Assessment

All citations were managed and organized using EndNote X9 software, and after duplicate records were removed, the two researchers made preliminary exclusions based on selection criteria by reading titles and abstracts. They then downloaded the full text of the remaining literatures and extracted the following data: 1) Publication information: first author and year of publication; 2) Patient characteristics: Age, number, sex, tumor stage, and Child-Pugh grade. 3) Intervention information: type of intervention, duration, and dose; 4) Outcome data: immune indicators, clinical effectiveness rate, KPS, tumor markers, and ADRs; and 5) Quality evaluation items: selection bias, performance bias, detection bias, attrition bias, reporting bias, and other biases. The above results were analyzed using Review Manager 5.3, and each deviation was assigned three levels: low risk, unclear, and high risk. If there is a disagreement during the evaluation process, a third investigator is invited to make the final judgment.

### Statistical Analysis

STATA 15.0 and Review Manager 5.3 were used for data analysis in this NMA 18. The results of the binary variables were evaluated as odds ratios (OR). Results of continuous variables were evaluated as the standard mean difference/mean difference (SMD/MD). When 95% confidence intervals (95% CIs) of OR value did not contain 1 and MD/SMD did not contain 0, it represented statistical difference. Heterogeneity was analyzed using Cochrane's Q test and quantified by I^2^ statistic. When p-value > 0.05, or I^2^ < 50% indicates low heterogeneity, the fixed effect model was used. Otherwise, heterogeneity was large, and random effect model should be adopted. We used Review Manager 5.3 to compare the outcomes of combined ST and TCM versus ST alone and to draw forest plots. STATA 15.0 was used to conduct NMA, and a frequency analysis framework was adopted to draw network relation graph, cumulative ranking graph 19. The results of network analysis were obtained by integrating direct and indirect comparison results, and publication bias was detected by Egger's test or comparison-adjusted funnel plots 20. Subgroup analysis, sensitivity analysis and meta-regression analysis were performed according to the drug type of ST, TCM efficacy, drug-delivery way, and course. Finally, cluster analysis of immune-related indexes was carried out.

## Results

### Baseline Characteristics of Included Studies

**Figure 2:**
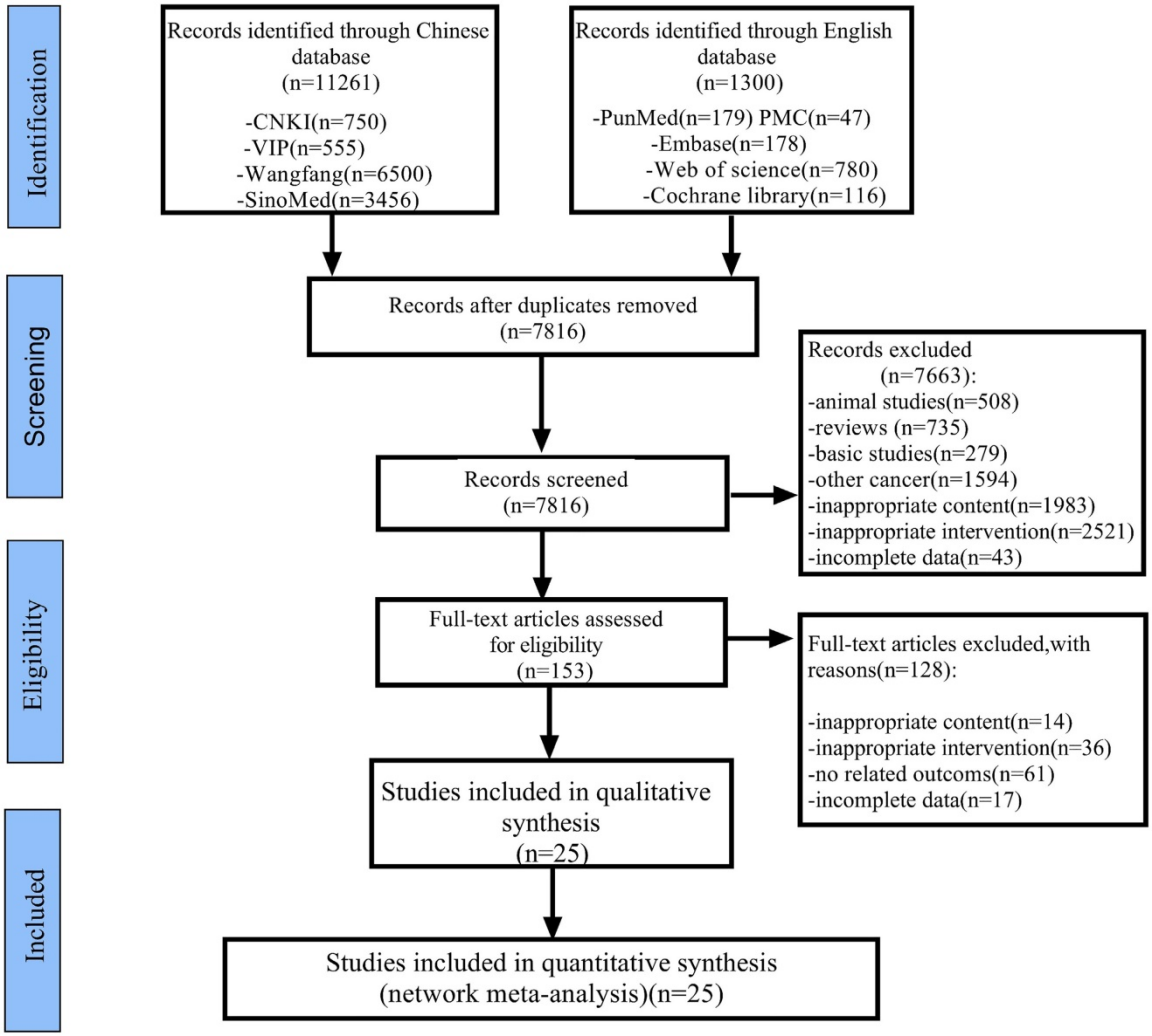
Flowchart of the study selection for the systematic review and network meta-analysis.

**Figure 3:**
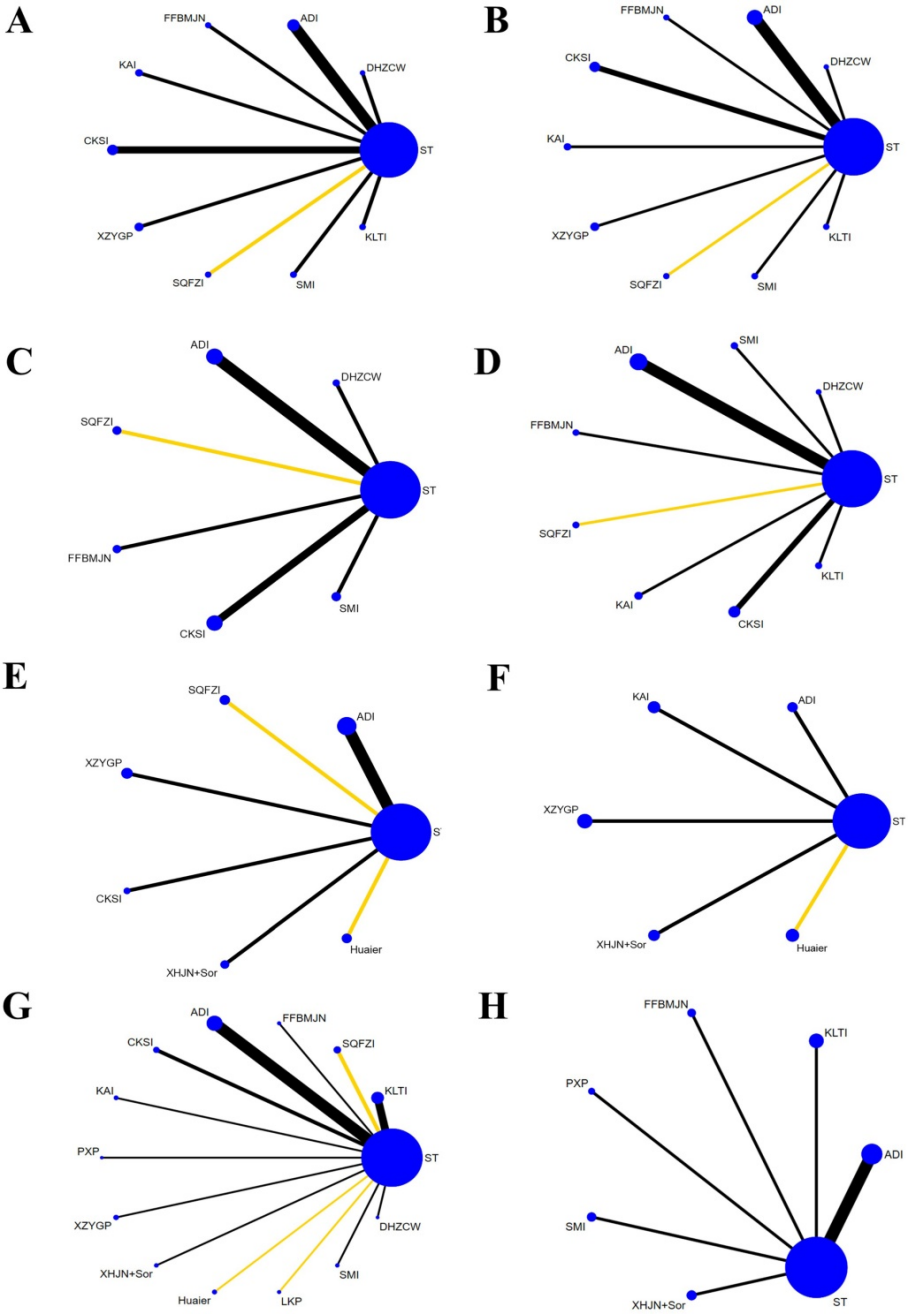
** Network graphs of different outcomes: A, CD3^+^; B, CD4^+^; C, CD8^+^; D, CD4^+^/CD8^+^; E, VEGF; F, AFP; G, Clinical effectiveness rate; H, Quality of Life.** Note: The yellow line: Sorafenib; The black line: systematic chemotherapy; VEGF: vascular endothelial growth factor; AFP: alpha fetoprotein; KLTI: Kanglaite injection; ADI: Aidi injection; FFBMJN: Fufang Banmao Jiaonang; PXP: Pingxiao Pian; KAI:Kangai injection; CKSI: compound kushen injection; SQFZI: Shenqifuzheng injection; LKP: Longkui Pian; SMI: Shenmai injection; XZYGP: Xiaozheng Yigan Pian; XHJN: Xihuang Jiaonang; Huaier: Huaier granule; Sor: sorafenib; ST: systemic therapy.

As shown in Figure 2, 12,561 articles were retrieved by applying the established retrieval strategy from electronic databases for the initial literature retrieval (including Chinese articles: 11,261; English articles: 1,300). After eliminating duplicate articles, 7,816 articles remained viable for analysis. According to the selection criteria established, 153 studies were retained after the titles and abstracts of the remaining papers were reviewed. Download the full text and after careful review, a total of 128 articles were excluded, for the following reasons: inappropriate content (n = 14), inappropriate intervention (n = 36), irrelevant outcomes (n = 61), and incomplete data (n = 17). Finally, 25 RCTs involving six types of CHIs and seven kinds of proprietary Chinese medicines were analyzed. CHIs were as follows: Aidi injection (ADI: eight trials), Kanglaite injection (KLTI: four trials), Shenqifuzheng injection (SQFZI: two trials), Kangai injection (KAI: one trial), compound kushen injection (CKSI: two trials), Shenmai injection (SMI: one trial), proprietary Chinese medicines: Dahuang Zhechong Wan (DHZCW: one trial), Fufang Banmao Jiaonang (FFBMJN: one trial), Pingxiao Pian (PXP: one trial), Xiaozheng Yigan Pian (XZYGP: one trial), Xihuang Jiaonang (XHJN: one trial), Huaier granule (Huaier: one trial), and Longkui Pian (LKP: one trial). According to the main effects of TCM, it can be divided into nourishing groups (including KAI, SQFZI, KLTI, SMI and Huaier) and attacking evil groups ((including ADI, CKSI, FFBMJN, PXP, XHJN, XZYGP, DHZCW and LKP). In addition, ST includes sorafenib (Sor) and systematic chemotherapy (CT), which can be divided into the following categories according to the composition of chemotherapy drugs: CAFI, FMEA, FAP, FOLFOX, XELOX, GP, PE, Arsenite injection, 5-fluorouracil, and others (Supplementary Material Document 3: Drug grouping and composition). All studies are from China. The characteristics of studies are shown in Table 1, and the network diagram is shown in Figure 3.

### Methodological Quality

To measure article quality, tools used by the Cochrane collaboration to assess the risk of bias in randomized trials were used 21. With regards to the selection bias, 12 of the 25 studies described the randomization methods, which included random number table or roll the dice, and were rated as low risk trials. One trial was randomized based on the order of admission and was considered to be a high-risk trial. The rest of the trials did not describe the process of randomization in detail, and the risk was considered unclear. None of the included studies described the allocation of the concealment scheme in detail. Therefore, the performance bias and selection bias were evaluated as unclear. Regarding the implementation of blinding, none of the included trials mentioned the implementation of blinding in detail and were therefore considered unclear. All trials were assessed as low risk in terms of data integrity, selective reporting, and other sources of bias (Supplementary Material Document 3: Risk of bias graph).

### Network Meta-Analysis

#### Immune-related indicators

The analysis of immune indices in this study mainly involved T-lymphocyte subsets (CD3^+^, CD4^+^, CD8^+^, and CD4^+^/CD8^+^) and VEGF levels.

**Figure 4:**
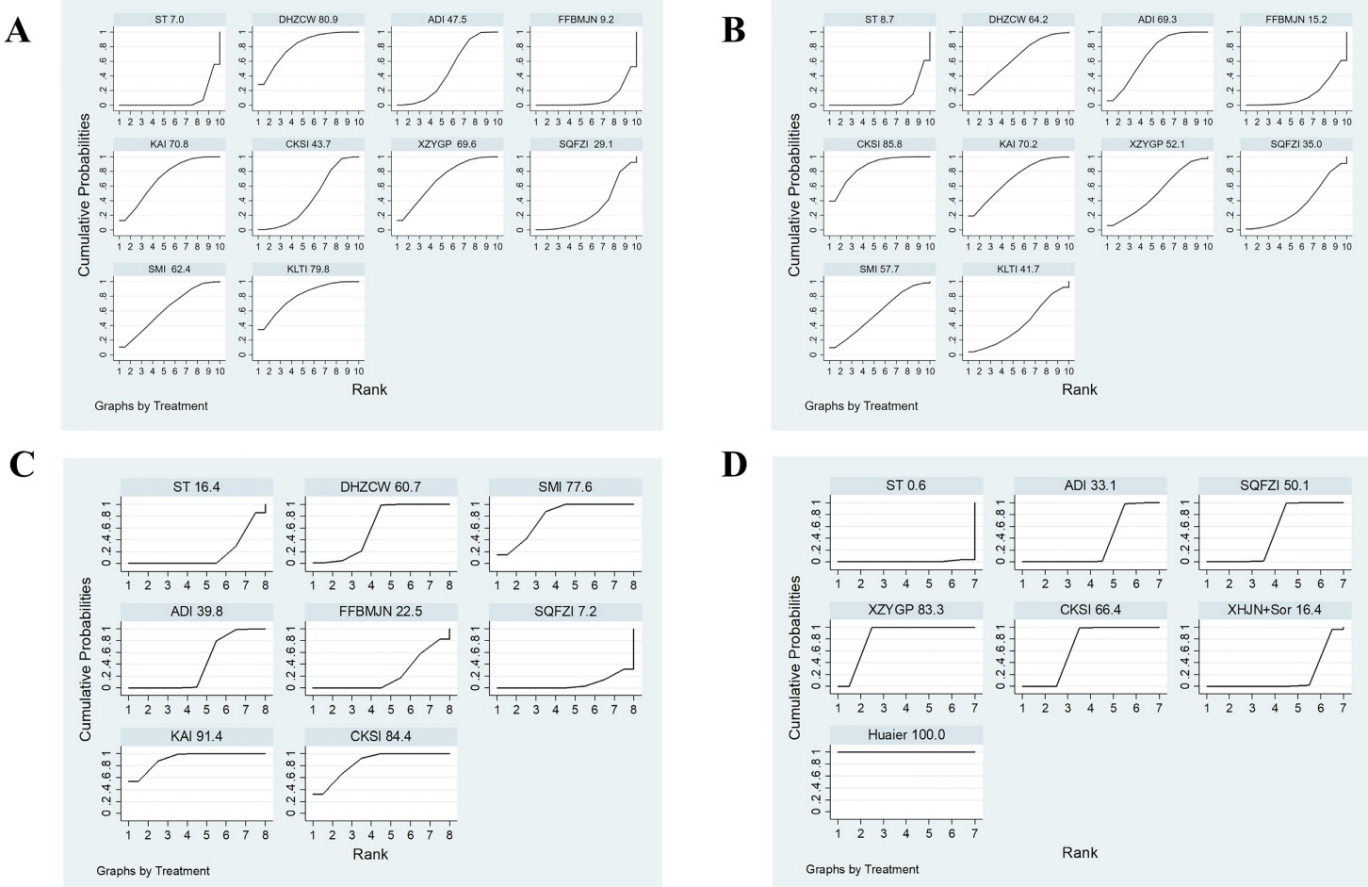
** Plot of the surface under the cumulative ranking curves for Immune-related indicators (A, CD3^+^; B, CD4^+^; C, CD4^+^/CD8^+^; D, VEGF).** Note: KLTI: Kanglaite injection; ADI: Aidi injection; FFBMJN: Fufang Banmao Jiaonang; PXP: Pingxiao Pian; KAI:Kangai injection; CKSI: compound kushen injection; SQFZI: Shenqifuzheng injection; LKP: Longkui Pian; SMI: Shenmai injection; XZYGP: Xiaozheng Yigan Pian; XHJN: Xihuang Jiaonang; Huaier: Huaier granule; Sor: sorafenib; ST: systemic therapy.

A total of 12 studies involved CD3^+^, and consisted of six kinds of CHIs, three kinds of proprietary Chinese medicines, and ten interventions. MD values and 95% CIs showed that combination therapy significantly increased CD3^+^ levels (7.69, 4.18 ~ 11.20) (Supplementary Material Document 3: Forest plot) and ST combined with DHZCW (12.25, 5.76~18.74), ADI (7.20, 3.38~11.02), KAI (10.49, 4.33~16.65), CKSI (6.65, 1.88~11.42), XZYGP (10.35, 3.68~17.02), SMI (9.29, 1.50~17.08), and KLTI (12.30, 4.13~20.47)could significantly increase the level of CD3^+^ in the patients compared to ST alone (Table 2). After ranking the interventions, the surface under the cumulative ranking curve (SUCRA) suggested that the first three interventions in the order of ranking were DHZCW (80.9%), KLTI (79.8%), and KAI (70.8%) (Figure 4A, Table 3).

There were 13 studies involving CD4^+^, which contained six kinds of CHIs, three kinds of proprietary Chinese medicines, and ten interventions. According to the MD values and 95% CIs, combination therapy significantly increased CD4^+^ levels (8.40, 4.52 ~ 12.28) (Supplementary Material Document 3: Forest plot) and ST combined with DHZCW (9.28, 1.04~17.52), ADI (9.89, 5.69~14.09), KAI (10.26, 2.44 ~18.08), and CKSI (12.59, 6.97~18.21) could significantly increase the level of CD4^+^ in the patients compared to ST alone (Table 2). The SUCRA indicated that the first three interventions in the order of ranking were CKSI (85.8%), KAI (70.2%), and ADI (69.3%) (Figure 4B, Table 3).

A total of nine studies involved CD8^+^ level, which investigated four kinds of CHIs and two kinds of proprietary Chinese medicines, and seven interventions. The MD values and 95% CIs showed that there was no significant difference in the improvement of CD8^+^ level by combination therapy (0.08, -2.31 ~ 2.47) (Supplementary Material Document 3: Forest plot). Nonetheless, NMA analysis revealed that compared with SMI combined with ST, ADI combined with ST had a significant advantage in improving CD8^+^ levels (14.55, 3.68 ~ 25.41) (Table 2).

There were 12 studies involving CD4^+^/ CD8^+^, which investigated six kinds of CHIs, two kinds of proprietary Chinese medicines, and nine interventions. According to the MD values and 95% CIs, combination therapy significantly increased CD4^+^/CD8^+^ levels (0.30, 0.16 ~ 0.45) (Supplementary Material Document 3: Forest plot) and ST combined with DHZCW (0.4, 0.18~0.62), ADI (0.13, 0.01~0.24), KAI (0.69, 0.48~0.90), CKSI (0.62, 0.36~0.88), SMI (0.56, 0.32~0.80), and KLTI (0.61, 0.26~0.96) could significantly improve the decrease in CD4^+^/ CD8^+^ level compared to ST alone (Table 2), while the SUCRA indicated that the first three interventions in the order of ranking were KAI (91.4%), CKSI (84.4%), and SMI (77.6%) (Figure 4C, Table 3).

A total of eight studies reported VEGF, which investigated three kinds of CHIs, three kinds of proprietary Chinese medicines, and seven interventions. According to the SMD values and 95% CIs, combination therapy significantly decreased VEGF levels (-2.07, -3.10 ~ -1.04) (Supplementary Material Document 3: Forest plot) and ST combined with CKSI (-2.28, -2.88 ~ -1.68), XZYGP (-4.16, -4.77 ~ -3.54), SQFZI (-1.48, -1.89 ~ -1.07), ADI (-0.85, -1.12 ~ -0.58) and Huaier (-6.05, -6.94~-5.17) significantly reduced VEGF levels compared to ST alone, and the SUCRA indicated that the first three interventions in the order of ranking were Huaier (100%), XZYGP (83.3%), and CKSI (66.4%) (Supplementary Material Document 3: NMA results of VEGF and AFP, Figure 4D).

At the same time, there were three studies involving CD3^+^/CD8^+^, two studies involving NK levels, three studies involving Intercellular Adhesion Molecule 1 ( ICAM-1) level, two involving IL-2 level, two involving IL-6 level, two involving transforming growth factor-β (TGF-β) level, and two involving tumor necrosis factor alpha (TNF-α) level, the MD values and 95% CIs for these groups of studies, respectively, were as follows: 0.5(0.44~0.56), 5.77(4.47~7.08), -122.91(-137.07 ~ -108.74), -4.36,(-17.45~8.74), -0.2(-0.22~-0.19), -14.05(-25.77~-2.33), and -0.84(-4.10~2.43). All the indicators improved to varying degrees after the treatment, and there were statistically significant differences among the four indicators of CD3^+^/CD8^+^, NK, ICAM-1, IL-6, and TGF-β (Supplementary Material Document 3: Forest plot).

#### Tumor markers (AFP)

A total of five studies involving AFP, including two CHIs, three proprietary Chinese medicines, and six interventions. According to the SMD values and 95% CIs, combination therapy significantly could significantly reduce AFP levels (-4.16, -5.59 ~ -2.74) and ST combined with Huaier (-2.42, -2.76 ~ -2.07), XHJN (-4.39, -5.13 ~ -3.64), XZYGP (-3.30, -3.83 ~ -2.77), KAI (-8.75, -10.01 ~ -7.50), and ADI (-2.51, -3.08 ~ -1.93) significantly reduced AFP levels compared to ST alone (Supplementary Material Document 3: Forest plot and NMA results of VEGF and AFP).

#### Clinical effectiveness rate

**Figure 5:**
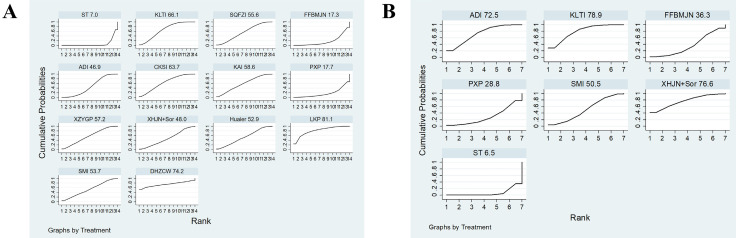
**Plot of the surface under the cumulative ranking curves (SUCRA) for A, Clinical effectiveness rate; B, Quality of Life.** Note: KLTI: Kanglaite injection; ADI: Aidi injection; FFBMJN: Fufang Banmao Jiaonang; PXP: Pingxiao Pian; KAI:Kangai injection; CKSI: compound kushen injection; SQFZI: Shenqifuzheng injection; LKP: Longkui Pian; SMI: Shenmai injection; XZYGP: Xiaozheng Yigan Pian; XHJN: Xihuang Jiaonang; Huaier: Huaier granule; Sor: sorafenib; ST: systemic therapy.

A total of 23 studies reported the results of clinical effectiveness, involving six CHIs, seven proprietary Chinese medicines, and 14 interventions. According to the OR and 95% ICs, combination therapy (2.54, 2.10 ~ 3.07) could effectively improve the clinical efficacy of patients (Supplementary Material Document 3: Forest plot) and ST combined with SMI 2.48(1.06~5.80), LKP 4.57(1.58~13.22), Huaier 2.50(1.16~5.38), XZYGP 2.74(1.31~5.72), KAI 2.74(1.25~6.03), CKSI 2.97(1.54~5.75), ADI 2.36(1.59~3.50), SQFZI 2.57(1.39~4.78), and KLTI 2.96(1.90~4.59) can significantly improve the clinical response rate compared to ST alone (Table 4). The SUCRA indicated that the first three interventions in the order of ranking were LKP (81.1%), DHZCW (74.2%), and KLTI (66.1%) (Figure 5A, Table 5).

#### Quality of life

A total of 9 studies reported performance status, which involved three CHIs, three proprietary Chinese medicines, and seven interventions. According to the OR values and 95% CIs combination therapy (2.87, 2.02 ~ 4.09) could effectively improve the Quality of life (Supplementary Material Document 3: Forest plot) and ST combined with KLTI (4.00, 2.03~7.89), ADI (3.50, 1.63~7.53), and XHJN (4.18, 1.25~13.94)) could significantly improve Quality of Life compared to ST alone (Table 4). The SUCRA indicated that the first three interventions in the order of ranking were KLTI (78.9%), XHJN (76.6%), and ADI (72.5%) (Figure 5B, Table 5).

#### ADRs

The major ADRs in this meta-analysis included three indicators: liver impairment, myelosuppression, and gastrointestinal reactions. At the same time, fever, pain, skin diseases, leukopenia, hair loss, thrombocytopenia, diarrhea, fatigue, and oral ulcer were also compared and analyzed.

Impaired liver function and bone marrow suppression were reported in seven separate studies, and 11 studies involved gastrointestinal reaction outcome indicators. According to the OR values and 95% CIs, it was found that combination therapy could effectively ameliorate ADRs (0.41, 0.35~0.49) and different TCM combination methods could significantly improve impaired liver function (0.40, 0.26~0.62), gastrointestinal reaction (0.51, 0.36~0.72), and myelosuppression (0.31, 0.21~0.46) compared with ST alone (Supplementary Material Document 3: Forest plot). The sequence of interventions suggested that KAI + ST had the highest likelihood of improving liver function impairment (74.4%) and gastrointestinal symptoms (86.7%), while CKSI + ST was the best choice for bone marrow suppression (92.8%) (Table 5).

We further investigated four studies involving fever, three involving pain, four involving skin diseases, three involving leukopenia, four reporting hair loss, two reporting thrombocytopenia, two reporting diarrhea, two involving fatigue, and two studies involving oral ulcers. ​The OR values indicated that in addition to pain and skin diseases, TCM combined with ST was better than ST alone in other adverse reactions (Supplementary Material Document 3: Forest plot).

In addition, the survival rate is an important index of response efficacy and improving the survival rate of patients is the ultimate goal of treatment. In our analysis, we investigated six studies involving survival results, and the analysis showed that compared with ST alone, the combination of TCM (OR: 1.70, 95%ICs: 0.86 ~3.36) did not significantly improve (P = 0.13) the half-year survival rate. However, the one-year overall survival rate of the combination of TCM treatment (OR: 3.00, 95%ICs: 1.85~4.86) was significantly higher than that of ST alone (P <0.05) (Supplementary Material Document 3: Forest plot).

#### Heterogeneity and publication bias

**Figure 6:**
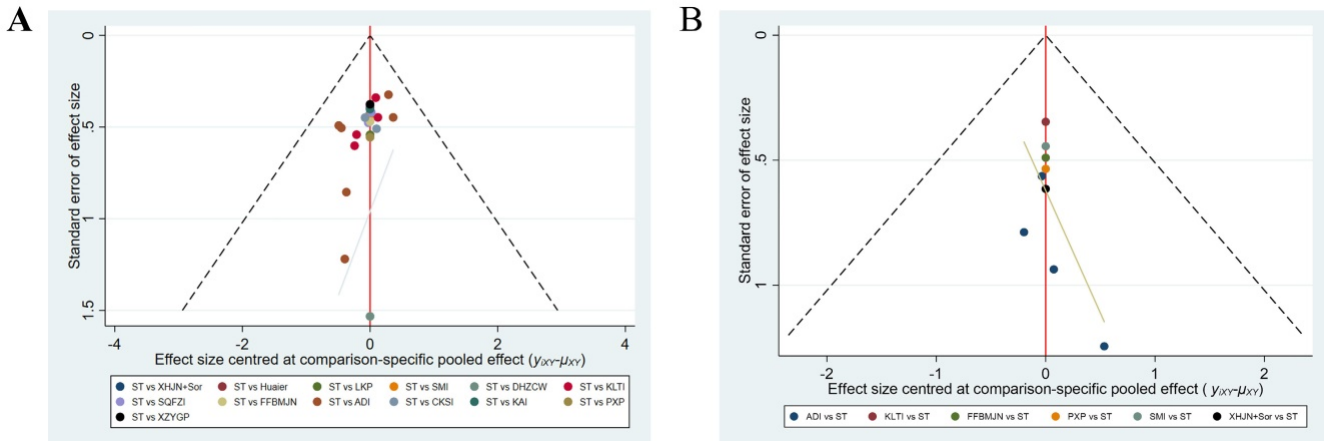
** Funnel plot of (A) clinical effectiveness rate and (B) Quality of Life.** Note: KLTI: Kanglaite injection; ADI: Aidi injection; FFBMJN: Fufang Banmao Jiaonang; PXP: Pingxiao Pian; KAI:Kangai injection; CKSI: compound kushen injection; SQFZI: Shenqifuzheng injection; LKP: Longkui Pian; SMI: Shenmai injection; XZYGP: Xiaozheng Yigan Pian; XHJN: Xihuang Jiaonang; Huaier: Huaier granule; Sor: sorafenib; ST: systemic therapy.

For results with heterogeneity (P < 0.05, I^2^ > 50), further analysis, immune-related indicators showed great heterogeneity overall. After subgroup analysis of ST types, heterogeneity was significantly improved. Combined with sensitivity analysis and meta-regression analysis, it was found that TCM efficacy, drug-delivery way and course were not the source of heterogeneity of the main outcome indicators (Table 6, Supplementary Material Document 3: Heterogeneity). There was no significant heterogeneity in clinical efficacy, quality of life and ADRs. Publication bias was determined by funnel plot and Egger's test, and most of the results showed no publication bias, and some results with bias were significantly improved after the subgroup analysis (Figure 6, Table 6).

#### Cluster analysis

**Figure 7:**
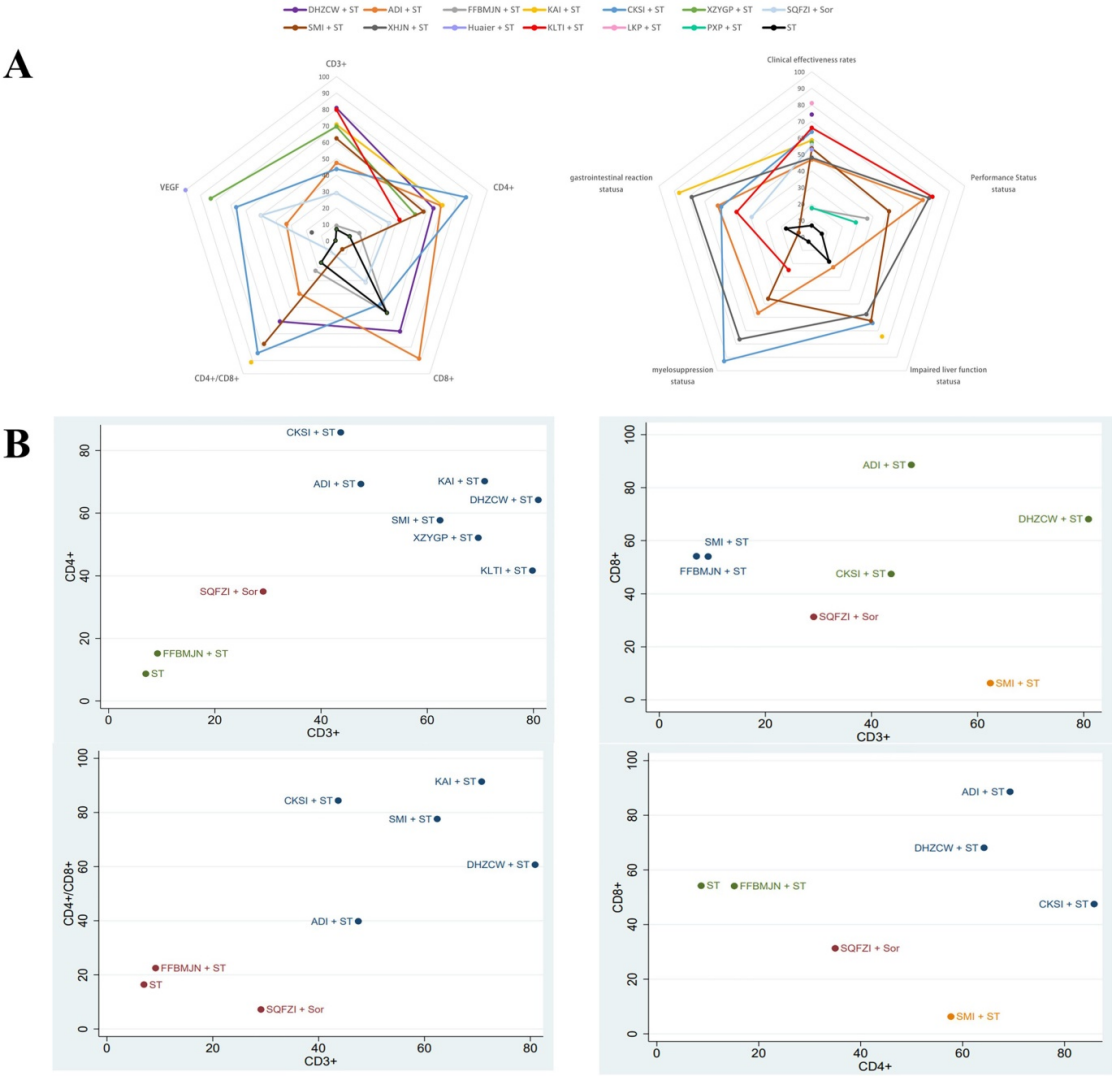
** Cluster analysis. A, The radar chart summarizes the comprehensive ranking status of the main indicators and secondary indicators, respectively, and the more external, the better the treatment; B, Main indexes were analyzed by pairwise clustering: CD3^+^ and CD4^+^, CD3^+^ and CD8^+^, CD3^+^ and CD4^+^/CD8^+^,CD4^+^ and CD8^+^, and measures of the same color in the figure have similar efficacy, and the closer they are to the upper right, the higher their overall ranking.** Note: KLTI: Kanglaite injection; ADI: Aidi injection; FFBMJN: Fufang Banmao Jiaonang; PXP: Pingxiao Pian; KAI:Kangai injection; CKSI: compound kushen injection; SQFZI: Shenqifuzheng injection; LKP: Longkui Pian; SMI: Shenmai injection; XZYGP: Xiaozheng Yigan Pian; XHJN: Xihuang Jiaonang; Huaier: Huaier granule; Sor: sorafenib; ST: systemic therapy.

Cluster analysis was conducted according to the SUCRA, and the overall ranking of each outcome was presented simply and clearly in the form of radar charts. At the same time, pairwise synthesis was performed for T-lymphocyte subsets in the main outcome to evaluate the relative best treatment method. Cluster analysis results showed that KAI and DHZCW are closest to the upper right, indicating that they have the best effect on improving CD3^+^ and CD4^+^ levels. DHZCW had the best effect on improving CD3^+^ and CD8^+^. ADI was most effective in improving CD4^+^ and CD8^+^. KAI was most effective in improving CD3^+^ and CD4^+^/CD8^+^. Overall, KAI and DHZCW had the best efficacy in combination ST to modulate immunity (Figure 7).

## Discussion

Liver cancer is a global health problem with increasing morbidity and mortality rates. Although the increasing use of surgical and local therapies worldwide, approximately 50-60% of patients will eventually receive ST^2^; however, the efficacy of ST has been unsatisfactory. In the early stage, CT is the primary treatment method for advanced liver cancer, but because of its strong drug resistance, resulting in a low benefit rate for patients. In recent years, with the research on molecular mechanisms of the tumor microenvironment (TME), it has been found that the abnormal regulation of various molecular mechanisms in the TME and the functional inhibition of immune-related cells are important factors that lead to the occurrence and progression of tumors 22. Simultaneously, it may be an important factor in the generation of CT drug resistance 23; subsequently, targeted therapies based on Sorafenib 24 and immunotherapies based on immune checkpoint inhibitors 25 have been developed, which achieved remarkable curative effects in clinical applications. More importantly, the combination immunotherapy regimen has emerged as a new first-line treatment. Thus far, ST has ushered in new prospects, and the combined scheme of ST focusing on regulating immunity is also being explored extensively. TCM has the characteristics of multiple approaches, multiple targets, and low side effects and can effectively regulate the immune microenvironment of tumors and increase therapeutic efficacy 26, 27. All kinds of proprietary Chinese medicines and CHIs have been widely used in clinical practice due to their fixed components, convenience, and rapidness. However, in the field of liver cancer, few studies have directly compared the immunomodulatory effects of their combination with ST, which makes it difficult for clinicians to select TCM with anti-tumor effect and good immunomodulatory effect at the same time. Therefore, we conducted an NMA to evaluate the efficacy of different types of proprietary Chinese medicines or CHIs in the combined ST to regulate the immune function of liver cancer patients.

According to our NMA analysis, compared with ST alone, the levels of CD3^+^, CD4^+^, CD3^+^/CD8^+^, CD4^+^/CD8^+^, and NK can be significantly increased by the combination of TCM and ST. In addition, through ranking, it was found that DHZCW, CKSI, and KAI combined with ST were the best ways to improve CD3^+^, CD4^+^, and CD4^+^/CD8^+^, respectively. Although CD8^+^ levels showed no statistically significant differences after NMA analysis, differences were observed in individual studies after subgroup analysis. Other meta-analysis have also shown that there is no unified conclusion on the changes of CD8^+^ level after TCM treatment 28, which is mainly may be the fact that the clinical detection of CD8^+^T cells is still limited to the overall number, without considering the subgroup status of CD8^+^T cells, because the co-inhibitory molecules on the surface of CD8^+^T cells (such as: PD-1,TIGIT, TIM3) leads to the exhaustion of some CD8^+^T cells function. If the total number of CD8^+^T cells is considered only, it may be impossible to accurately judge its influence on CD8^+^T cells. Our team's previous studies also confirmed that PD-1^+^ TIGIT^+^ CD8^+^ T-cell populations, which show co-inhibitory molecules, have shown functional exhaustion, which is related to disease progression 29. Therefore, attention should be paid to the classification of subgroups in future clinical tests, which will help us better analyze the immune status of patients. In addition, based on cluster analysis, KAI and DHZCW appeared to have the best potential for improving immunity in patients.

Intrahepatic NK cells play a central role in innate immune responses to liver pathogens and tumors 30. More importantly, T lymphocyte mediated cellular immunity plays an important role in the tumor microenvironment. T lymphocytes can be divided into various subtypes according to different surface markers. CD3 is a common surface marker of T cells, and often forms the T cell receptor (TCR)/CD3 complex to participate in T cell recognition of antigen and signal transduction 31. CD3^+^ is represented as mature T lymphocytes. Based on this, CD4^+^T cells are helper T cells, which can secrete cytokines and activate CD8^+^T cells to carry out anti-tumor effects and are the most important hub cells in adaptive immune response. CD8^+^T cells are killer T cells that can directly kill tumor cells by releasing perforin and granzymes. CD4/CD8 is an important clinical index to judge the balance of immune system 32. These surface molecules are often measured clinically to measure the immune function of patients and become an important indicator for monitoring tumor development and predicting clinical therapeutic effect and prognosis.

Studies have shown that the development of liver cancer depends on the formation of chronic inflammation and immune escape 33, so it is very important to anti-inflammatory and reverse the escape. Animal experiments have shown that: DHZCW can induce tumor cell apoptosis by reversing the balance of Treg/TH1, which are derived from naive CD4^+^T cells, but one of them has a negative regulation on immunity, while the other has a positive effect on immunity. By inhibiting the differentiation of naive CD4^+^T cells into Treg and promoting their differentiation into TH1, the suppressed immune state of the body can be improved 34. According to pharmacological analysis, the main components of DHZCW include rhein and baicalin, etc. Rhein can inhibit the production of pro-inflammatory factors (IL-6 and IL-1β) by inhibiting the level of NF-κB, thus exerting anti-inflammatory effect 35. Baicalin reduced STAT3 activity, further down-regulated IFN-γ -induced PD-L1 expression, and subsequently restored T cell sensitivity to kill tumor cells, thereby reversing immune escape 36. KAI is a commonly used TCM for the treatment of tumors. Studies have shown that KAI can inhibit the proliferation of cancer cells by inhibiting IL-6/ STAT3 and play an anti-tumor role 37. Pharmacological analysis showed that its main active ingredients were astragaloside, ginsenoside, oxymatrine, etc. 38. Multiple studies have shown that these ingredients could enhance the expression of CD8^+^T cells and NK cells, promote the infiltration of CD4^+^T cells and CD8^+^T cells and inhibit inflammation to enhance the anti-tumor effect of the body 39-41. The main components of CKSI are matrine and hydroxymatrine, which exert a variety of pharmacological activities 42 that include anti-inflammatory effects, inhibition of angiogenesis and cancer metastasis and invasion, reversing multi-drug resistance, preventing, or reducing the toxicity induced by chemotherapy or therapy. At the same time, it can also regulate serum IL-2, IL-4, IL-10, IL-6 and TNF-α levels and improving immunity activity 43. In terms of the efficacy of the TCM, although KAI and DHZCW have different efficacy (one focuses on nourishing, while the other is good at attacking), both can effectively regulate T-cell immunity and reduce inflammation. It is widely believed that nourishing Chinese medicine can improve the immunity of patients to fight cancer, while attacking drugs are mainly to directly kill cancer cells to achieve the purpose of anti-tumor 26. Our results may suggest that Chinese medicines with different therapeutic effects can effectively regulate immunity, which is consistent with the results of this subgroup analysis. Because the sample size is small and the quality of evidence is not high, to follow-up extensive clinical trials are still required to confirm this conclusion. In addition, VEGF 44, ICAM-1 45-47, TGF-β 48, IL-2, IL-6^49^-^51^, and TNF-α 52 play an important role in the growth and metastasis of tumor cells, and can affect the expression of co-inhibitory molecules on immune cells. In this NMA study, compared with ST alone, combined TCM can significantly reduce the expression of VEGF, ICAM-1, IL-6, and TGF-β levels, thus improving the immunity of liver cancer patients. Huaier granule has the best effect on reducing the VFGF level. Its main ingredient, Huaier polysaccharide, can increase the proportion of CD4^+^ T cells and NK cells by promoting the secretion of cytokines IL-2 and IFN-γ and inhibiting the secretion of immunosuppressive serum cytokine IL-10 53. TCM combined with ST also greatly improved the level of AFP, clinical effectiveness rate, Quality of Life, survival rate, and ADRs, indicating that TCM combined with ST can also significantly improve the therapeutic effect and safety of treatment. It is worth noting that KAI and CKSI have a better ability to improve ADRs than other drugs.

However, this NMA also has some limitations. First, the 25 trials were all from China, and the TNM tumor staging of patients were different, which may have led to heterogeneity within the results. Second, the number of RCTs included was small, none of the studies mentioned the procedure of blindness and allocation hiding, and the number of RCTs involved in each intervention was different. Third, due to the limited number of studies, the indicators for measuring immune function were not complete and most of the indicators were only from a single study, which may directly lead to lower reliability of the results. Therefore, we suggest that large-sample, multi-center, high-quality randomized controlled trials should be included in the future to support our view.

## Conclusion

Overall, current evidence suggests that TCM combined with ST may be more beneficial than ST alone in improving immune function in liver cancer patients, and DHZCW and KAI combined with ST group had a higher advantage in regulating immunity. The main mechanism of its action may be regulating cytokine secretion, inhibiting inflammatory response, and reversing functional depletion of immune cells. However, due to the limitations of this study, more multicenter, large-sample RCTs are needed to support the results of this NMA study.

## Supplementary Material

Supplementary materials.

## Figures and Tables

**Table 1:** Basic characteristics of the eligible studies

Study ID	Cases	KPS score	TNM clinical stage (E/C)				Treatment group intervention	Control group intervention	Course	Outcomes
	(E/C)	(E/C)	Ⅰ	Ⅱ	Ⅲ	Ⅳ				
Yang et al.(2018)^54^	35/30	NR	Ⅱb-Ⅲa				DHZCW+ CT (CAFI)	CT (CAFI)	14d	①②④⑥
Wang CZ(2016)^55^	34/34	NR	NR				ADI 50ml + CT (CAFI)	CT (CAFI)	28d	①②③④
Lv et al.(2019)^56^	27/27	>60	NR				KLTI 200mL +CT (FMEA)	CT (FMEA)	20d	①②③④
Li et al.(2014)^57^	75/75	NR	28	32	59	31	KLTI 200mL + CT (FMEA)	CT (FMEA)	20d	①②③④⑥
Kang et al. (2021)^58^	43/43	NR	10/11	9/10	16/14	8/8	KLTI 200mL + CT (FMEA)	CT (FMEA)	20d	①②
Xiao et al.(2012)^59^	30/30	≥50	/	12/13	18/17	/	PXP + CT (FAP)	CT (FAP)	26d*2	①②③
Wang et al. (2015)^60^	79/77	NR	26/21	38/36	15/20	/	ADI 60ml+ CT (FOLFOX)	CT (FOLFOX)	28d*4	①②
Tang Mi. (2021)^61^	53/53	NR	/	8/6	28/29	17/18	KAI 40 ml + CT (FOLFOX)	CT (FOLFOX)	28d*3	①②③④⑤
Tang HB. (2016)^62^	45/45	≥ 60	/	/	24/26	21/19	SMI 60ml + CT (FOLFOX)	CT (FOLFOX)	15d*2	①②③④
Ma et al.(2017)^63^	43/43	NR	NR				KLTI 200ml + CT (FOLFOX)	CT (FOLFOX)	28d	①②③
Teng et al. (2020)^64^	65/65	≥70	/	/	56/58	9/7	XZYGP + CT (FOLFOX)	CT (FOLFOX)	14d*6	①②⑤
Han Yu.(2020)^65^	37/37	NR	NR				ADI 50ml+ CT (XELOX)	CT (XELOX)	14d*4	①②③
Zhang MM.(2018)^66^	35/35	NR	NR				ADI 50ml+ CT (XELOX)	CT (XELOX)	14d*4	①②③
Zhang et al. (2018)^67^	36/36	≥60	/	7/8	19/20	10/8	CKSI 30ml + CT (GP)	CT (GP)	28d*2	①②④
Zhang and Zhou.(2014)^68^	48/42	≥60	Ⅲb - Ⅳ				CKSI 30ml + CT (GP)	CT (GP)	28d*2	①②④
Shi and Wang (2011)^69^	35/35	NR	NR				ADI 100ml+ CT (PE)	CT (PE)	20d*2	①②③⑤⑥
Li RC. (2012)^70^	15/15	NR	NR				ADI 80ml+ CT (PE)	CT (PE)	20d*2	①②③⑤⑥
Hou et al.(2017)^71^	43/43	>60	/	9/11	21/18	13/14	ADI 50ml+ CT (5-fluorouracil)	CT (5-fluorouracil)	30d	①②③⑤
Weng et al.(2010)^72^	19/18	40-70	NR				ADI 50ml +CT (Arsenite injection)	CT (Arsenite injection)	28d	①③④⑤
Peng WD. (2011)^73^	40/40	≥50	/	17/15	23/25	/	FFBMJN + CT (FAP)	CT (FAP)	26d*2	①②③
Feng et al. (2020)^74^	49/48	NR	/	20/18	29/30	/	XHJN + Sor+ CT (other)	CT (other)	2m	①②③④⑤
Tang et al. (2018)^75^	57/56	NR	NR				Huaier + Sor	Sor	2m	①②③④⑤⑥
Hao WJ. (2020)^76^	42/42	NR	/	/	19/20	23/22	SQFZI 250ml + Sor	Sor	21d*2	①②
Yang et al.(2017)^77^	41/41	NR	9/10	16/15	16/16	/	LKP + Sor	Sor	3m	①②⑥
Liu et al.(2019)^78^	58/58	>60	/	/	31/28	27/30	SQFZI 250ml + Sorafenib	Sor	21d*2	①②④

**Note**: ①immune-related indicators; ②the clinical effectiveness rate; ③KPS; ④adverse drug events (ADRs); ⑤AFP; ⑥Survival rates. m: month; d: day; E, Experimental group; C, Control group; CT, systematic chemotherapy; Sor, Sorafenib; TNM, tumor-node-metastasis; NR, not reported; AFP: alpha fetoprotein; KLTI: Kanglaite injection; ADI: Aidi injection; FFBMJN: Fufang Banmao Jiaonang; PXP: Pingxiao Pian; KAI: Kangai injection; CKSI: compound kushen injection; SQFZI: Shenqifuzheng injection; LKP: Longkui Pian; SMI: Shenmai injection; XZYGP: Xiaozheng Yigan Pian; XHJN: Xihuang Jiaonang; Huaier: Huaier granule; FMEA: fluorouracil + doxorubicin + semustine; CAFI: cisplatin + fluorouracil + azithromycin + interferon; FAP: fluorouracil + doxorubicin + cisplatin; FOLFOX: oxaliplatin + leucovorin + fluorouracil; XELOX: capecitabine + oxaliplatin; PE: pharmorubicin + cisplatin; GP: gemcitabine + cisplatin; other: fluorouracil, cytoxan...

**Table 2:** Results of the network meta-analysis for main outcomes (MD value, 95% CI)

		CD3^+^		CD4^+^		CD8^+^		CD4^+^/CD8^+^
**DHZCW + ST**	**vs**							
ADI + ST		5.05 (-2.48,12.58)		-0.61 (-9.85,8.64)		-2.76 (-13.60,8.08)		**0.27 (0.03,0.52)**
FFBMJN + ST		**12.28 (3.41,21.15)**		8.73 (-2.55,20.01)		2.20 (-10.67,15.07)		**0.37 (0.08,0.66)**
KAI + ST		1.76 (-7.19,10.71)		-0.98 (-12.34,10.38)		-		-0.29 (-0.59,0.01)
CKSI + ST		5.60 (-2.45,13.65)		-3.31 (-13.28,6.66)		2.94 (-8.39,14.27)		-0.22 (-0.56,0.12)
XZYGP + ST		1.90 (-7.40,11.20)		2.01 (-9.42,13.44)		-		-
SQFZI + ST		8.15 (-1.02,17.32)		4.80 (-6.58,16.18)		5.84 (-7.13,18.81)		**0.47 (0.17,0.77)**
SMI + ST		2.96 (-7.17,13.09)		1.10 (-10.74,12.94)		11.79 (-1.47,25.05)		-0.16 (-0.49,0.17)
KLTI + ST		-0.05 (-10.48,10.38)		3.78 (-8.45,16.01)		-		-0.21 (-0.62,0.20)
ST		**12.25 (5.76,18.74)**		**9.28 (1.04,17.52)**		2.11 (-7.25,11.47)		**0.40 (0.18,0.62)**
**ADI + ST**	**vs**							
FFBMJN + ST		**7.23 (0.07,14.39)**		**9.34 (0.56,18.11)**		4.96 (-5.44,15.35)		0.10 (-0.13,0.32)
KAI + ST		-3.29 (-10.54,3.96)		-0.37 (-9.25,8.50)		-		**-0.56 (-0.80, -0.33)**
CKSI + ST		0.55 (-5.57,6.67)		-2.70 (-9.72,4.31)		5.70 (-2.71,14.11)		**-0.49 (-0.78, -0.21)**
XZYGP + ST		-3.15 (-10.84,4.54)		2.62 (-6.36,11.59)		-		-
SQFZI + ST		3.10 (-4.42,10.62)		5.41 (-3.49,14.31)		8.60 (-1.92,19.12)		0.20 (-0.04,0.43)
SMI + ST		-2.09 (-10.77,6.58)		1.71 (-7.78,11.19)		**14.55 (3.68,25.41)**		**-0.43 (-0.70, -0.17)**
KLTI + ST		-5.10 (-14.12,3.92)		4.39 (-5.58,14.35)		-		**-0.48 (-0.85, -0.12)**
ST		**7.20 (3.38,11.02)**		**9.89 (5.69,14.09)**		4.87 (-0.61,10.34)		**0.13 (0.01,0.24)**
**FFBMJN + ST**	**vs**							
KAI + ST		**-10.52 (-19.16, -1.88)**		-9.71 (-20.68,1.26)		-		**-0.66 (-0.94, -0.38)**
CKSI + ST		-6.68 (-14.39,1.03)		**-12.04 (-21.57, -2.51)**		0.74 (-10.16,11.64)		**-0.59 (-0.92, -0.26)**
XZYGP + ST		**-10.38 (-19.39, -1.37)**		-6.72 (-17.77,4.33)		-		-
SQFZI + ST		-4.13 (-13.00,4.74)		-3.93 (-14.92,7.06)		3.64 (-8.96,16.24)		0.10 (-0.19,0.39)
SMI + ST		-9.32 (-19.19,0.55)		-7.63 (-19.10,3.84)		9.59 (-3.30,22.48)		**-0.53 (-0.84, -0.22)**
KLTI + ST		**-12.33 (-22.50, -2.16)**		-4.95 (-16.83,6.93)		-		**-0.58 (-0.98, -0.18)**
ST		-0.03 (-6.09,6.03)		0.55 (-7.15,8.25)		-0.09 (-8.93,8.75)		0.03 (-0.17,0.23)
**KAI + ST**	**vs**							
CKSI + ST		3.84 (-3.95,11.64)		-2.33 (-11.95,7.30)		-		0.07 (-0.26,0.40)
XZYGP + ST		0.14 (-8.94,9.22)		2.99 (-8.14,14.12)		-		-
SQFZI + ST		6.39 (-2.55,15.33)		5.78 (-5.30,16.86)		-		**0.76 (0.47,1.05)**
SMI + ST		1.20 (-8.73,11.13)		2.08 (-9.47,13.63)		-		0.13 (-0.19,0.45)
KLTI + ST		-1.81 (-12.04,8.42)		4.76 (-7.19,16.71)		-		0.08 (-0.33,0.49)
ST		**10.49 (4.33,16.65)**		**10.26 (2.44,18.08)**		-		**0.69 (0.48,0.90)**
**CKSI + ST**	**vs**							
XZYGP + ST		-3.70 (-11.90,4.50)		5.32 (-4.40,15.04)		-		-
SQFZI + ST		2.55 (-5.50,10.59)		8.11 (-1.54,17.76)		2.90 (-8.12,13.92)		**0.69 (0.36,1.02)**
SMI + ST		-2.64 (-11.78,6.49)		4.41 (-5.78,14.60)		8.85 (-2.50,20.20)		0.06 (-0.30,0.42)
KLTI + ST		-5.65 (-15.11,3.81)		7.09 (-3.55,17.73)		-		0.01 (-0.43,0.45)
ST		**6.65 (1.88,11.42)**		**12.59 (6.97,18.21)**		-0.83 (-7.21,5.55)		**0.62 (0.36,0.88)**
**XZYGP + ST**	**vs**							
SQFZI + ST		6.25 (-3.05,15.55)		2.79 (-8.37,13.95)		-		-
SMI + ST		1.06 (-9.19,11.31)		-0.91 (-12.54,10.72)		-		-
KLTI + ST		-1.95 (-12.50,8.60)		1.77 (-10.25,13.79)				-
ST		**10.35 (3.68,17.02)**		7.27 (-0.66,15.20)		-		-
**SQFZI + ST**	**vs**							
SMI + ST		-5.19 (-15.32,4.94)		-3.70 (-15.27,7.87)		5.95 (-7.04,18.94)		**-0.63 (-0.95, -0.31)**
KLTI + ST		-8.20 (-18.63,2.23)		-1.02 (-12.99,10.95)		-		**-0.68 (-1.09, -0.27)**
ST		4.10 (-2.38,10.58)		4.48 (-3.37,12.33)		-3.73 (-12.71,5.25)		-0.07 (-0.28,0.14)
**SMI + ST**	**vs**							
KLTI + ST		-3.01 (-14.30,8.28)		2.68 (-9.73,15.09)		-		-
ST		**9.29 (1.50,17.08)**		8.18 (-0.32,16.68)		**-9.68 (-19.07, -0.29)**		**0.56 (0.32,0.80)**
KLTI + ST	vs							
ST		**12.30 (4.13,20.47)**		5.50 (-3.54,14.54)		-		**0.61 (0.26,0.96)**

**Note**: underlined and bold values indicate statistical differences between groups. MD: mean difference; KLTI: Kanglaite injection; ADI: Aidi injection; FFBMJN: Fufang Banmao Jiaonang; KAI:Kangai injection; CKSI: compound kushen injection; SQFZI: Shenqifuzheng injection; SMI: Shenmai injection; XZYGP: Xiaozheng Yigan Pian; ST: systemic therapy.

**Table 3:** Surface under the cumulative ranking probabilities (SUCRA) results of immune-related indicators

Intervention	CD3^+^			CD4^+^			CD4^+^/CD8^+^	
	SUCRA(%)	Rank		SUCRA(%)	Rank		SUCRA(%)	Rank
DHZCW + ST	80.9	1		64.2	4		60.7	4
ADI + ST	47.5	6		69.3	3		39.8	5
FFBMJN + ST	9.2	9		15.2	9		22.5	6
KAI + ST	70.8	3		70.2	2		91.4	1
CKSI + ST	43.7	7		85.8	1		84.4	2
XZYGP + ST	69.6	4		52.1	6		-	-
SQFZI + Sor	29.1	8		35	8		7.2	8
SMI + ST	62.4	5		57.7	5		77.6	3
KLTI + ST	79.8	2		41.7	7		-	-
ST	7	10		8.7	10		16.4	7

**Note**: SUCRA was used to assess therapeutic efficacy, with higher SUCRA indicating better efficacy. SUCRA: the surface under the cumulative ranking curve; KLTI: Kanglaite injection; ADI: Aidi injection; FFBMJN: Fufang Banmao Jiaonang; KAI:Kangai injection; CKSI: compound kushen injection; SQFZI: Shenqifuzheng injection; SMI: Shenmai injection; XZYGP: Xiaozheng Yigan Pian; ST: systemic therapy.

**Table 4:** Results of the network meta-analysis for Clinical effectiveness (upper right) rate and Quality of Life (lower left) (OR value, 95% CI)

DHZCW	0.38 (0.02,8.57)	0.69 (0.03,16.82)	0.38 (0.02,8.45)	0.35 (0.02,7.92)	0.42 (0.02,9.19)	0.18 (0.01,4.34)	0.46 (0.02,9.95)	0.45 (0.02,9.80)	0.36 (0.02,7.42)	0.19 (0.01,4.39)	0.39 (0.02,8.41)	0.43 (0.02,9.03)	0.15 (0.01,3.07)
	**SMI**	1.84 (0.47,7.18)	1.01 (0.32,3.17)	0.93 (0.28,3.06)	1.10 (0.36,3.40)	0.47 (0.12,1.87)	1.22 (0.42,3.53)	1.20 (0.41,3.51)	0.95 (0.37,2.42)	0.50 (0.14,1.76)	1.04 (0.36,2.97)	1.14 (0.43,3.04)	**0.40 (0.17,0.94)**
		**LKP**	0.55 (0.15,2.03)	0.50 (0.13,1.95)	0.60 (0.16,2.19)	0.26 (0.06,1.17)	0.66 (0.19,2.29)	0.65 (0.19,2.27)	0.52 (0.17,1.60)	0.27 (0.07,1.11)	0.56 (0.16,1.93)	0.62 (0.19,1.99)	**0.22 (0.08,0.63)**
			**Huaier**	0.92 (0.30,2.87)	1.10 (0.38,3.17)	0.47 (0.12,1.77)	1.21 (0.45,3.28)	1.19 (0.43,3.27)	0.94 (0.40,2.23)	0.50 (0.15,1.65)	1.03 (0.38,2.76)	1.13 (0.46,2.80)	**0.40 (0.19,0.86)**
	0.56 (0.13,2.49)			**XHJN+Sor**	1.19 (0.39,3.64)	0.51 (0.13,2.01)	1.32 (0.46,3.78)	1.29 (0.44,3.76)	1.02 (0.40,2.59)	0.54 (0.16,1.88)	1.12 (0.39,3.17)	1.23 (0.47,3.24)	0.43 (0.19,1.01)
					**XZYGP**	0.43 (0.11,1.59)	1.11 (0.42,2.93)	1.09 (0.40,2.92)	0.86 (0.37,1.98)	0.46 (0.14,1.48)	0.94 (0.36,2.46)	1.03 (0.43,2.50)	**0.37 (0.17,0.76)**
	1.54 (0.39,6.01)			2.73 (0.55,13.48)		**PXP**	2.60 (0.74,9.17)	2.55 (0.71,9.10)	2.02 (0.63,6.43)	1.07 (0.26,4.45)	2.21 (0.63,7.72)	2.43 (0.74,7.99)	0.86 (0.29,2.55)
							**KAI**	0.98 (0.39,2.44)	0.78 (0.37,1.64)	0.41 (0.13,1.25)	0.85 (0.35,2.05)	0.93 (0.42,2.07)	**0.33 (0.17,0.62)**
								**CKSI**	0.79 (0.37,1.71)	0.42 (0.14,1.30)	0.87 (0.35,2.14)	0.95 (0.42,2.16)	**0.34 (0.17,0.65)**
	0.67 (0.21,2.14)			1.19 (0.29,4.98)		0.44 (0.12,1.60)			**ADI**	0.53 (0.19,1.44)	1.09 (0.52,2.28)	1.20 (0.64,2.24)	**0.42 (0.29,0.63)**
	1.31 (0.36,4.78)			2.32 (0.50,10.83)		0.85 (0.21,3.52)			1.94 (0.57,6.64)	**FFBMJN**	2.06 (0.68,6.26)	2.27 (0.80,6.43)	0.80 (0.32,2.01)
											**SQFZI**	1.10 (0.50,2.41)	**0.39 (0.21,0.72)**
	0.59 (0.20,1.77)			1.04 (0.26,4.16)		0.38 (0.11,1.33)			0.87 (0.31,2.44)	0.45 (0.14,1.46)		**KLTI**	**0.35 (0.22,0.57)**
	2.35 (0.99,5.62)			**4.18 (1.25,13.94)**		1.53 (0.54,4.36)			**3.50 (1.63,7.53)**	1.80 (0.69,4.70)		**4.00 (2.03,7.89)**	**ST**

**Note**: underlined and bold values indicate statistical differences between groups. KLTI: Kanglaite injection; ADI: Aidi injection; FFBMJN: Fufang Banmao Jiaonang; PXP: Pingxiao Pian; KAI:Kangai injection; CKSI: compound kushen injection; SQFZI: Shenqifuzheng injection; LKP: Longkui Pian; SMI: Shenmai injection; XZYGP: Xiaozheng Yigan Pian; XHJN: Xihuang Jiaonang; Huaier: Huaier granule; ST: systemic therapy.

**Table 5:** Surface under the cumulative ranking probabilities (SUCRA) results of secondary outcomes

Intervention	Clinical effectiveness rates		Quality of Life		Impaired liver function		myelosuppression		gastrointestinal reaction	
	SUCRA (%)	Rank	SUCRA (%)	Rank	SUCRA (%)	Rank	SUCRA (%)	Rank	SUCRA (%)	Rank
DHZCW + ST	74.2	2	-	-	-	-	-	-	-	-
ADI + ST	46.9	11	72.5	3	22.6	5	56.7	3	61.5	3
FFBMJN + ST	17.3	13	36.3	5	-	-	-	-	-	-
KAI + ST	58.6	5	-	-	74.4	1	-	-	86.7	1
CKSI + ST	63.7	4	-	-	64.3	2	92.8	1	59.3	4
XZYGP + ST	57.2	6	-	-	-	-	-	-	-	-
SQFZI + Sor	55.6	7	-	-	-	-	-	-	39.3	6
SMI + ST	53.7	8	50.5	4	62.7	3	46.1	4	8.6	8
XHJN + ST	48	10	76.6	2	57.7	4	76.4	2	78.5	2
Huaier + ST	52.9	9	-	-	-	-	-	-	-	-
KLTI + ST	66.1	3	78.9	1	-	-	24.6	5	49.2	5
LKP + ST	81.1	1	-	-	-	-	-	-	-	-
PXP + ST	17.7	12	28.8	6	-	-	-	-	-	-
ST	7	14	6.5	7	18.3	6	3.4	6	16.8	7

**Note**: SUCRA was used to assess therapeutic efficacy, with higher SUCRA indicating better efficacy. SUCRA: the surface under the cumulative ranking curve; KLTI: Kanglaite injection; ADI: Aidi injection; FFBMJN: Fufang Banmao Jiaonang; PXP: Pingxiao Pian; KAI:Kangai injection; CKSI: compound kushen injection; SQFZI: Shenqifuzheng injection; LKP: Longkui Pian; SMI: Shenmai injection; XZYGP: Xiaozheng Yigan Pian; XHJN: Xihuang Jiaonang; Huaier: Huaier granule; ST: systemic therapy.

**Table 6:** Summary of heterogeneity and publication bias regarding the outcomes of Immune-related indicators and AFP

Outcomes	Subgroups	MD/SMD (95%CI)	Heterogeneity	Metaregression	Publication bias (Egger's)
**CD3**	**Entire (12)**	7.69 [4.18, 11.20] *	P < 0.00001; I² = 98%†		p= 0.014*
	**CT (11):**	8.03 [4.22, 11.84] *	P < 0.00001; I² = 98%†	*** p= 0.678***	p=0.016*
	FOLFOX (4)	10.48 [9.37, 11.59] *	P = 0.88; I² = 0%		***p=0.889***
	GP (2)	6.19 [4.38, 7.99] *	P = 0.37; I² = 0%		
	PE (2)	4.79 [2.76, 6.82] *	P = 1.00; I² = 0%		
**CD4**	**Entire (13)**	8.40 [4.52, 12.28] *	P < 0.00001; I² = 99%†		p=0*
	**CT (12):**	8.74 [4.38, 13.09] *	P < 0.00001; I² = 99%†	***p= 0.585***	p=0.001*
	FOLFOX (4)	8.27 [6.14, 10.41] *	P = 0.03; I² = 66%†		***p=0.288***
	GP (2)	12.66 [11.30, 14.02] *	P = 0.49; I² = 0%		
	PE (2)	8.47 [6.33, 10.61] *	P = 1.00; I² = 0%		
	CAFI(2)	7.47 [3.33, 11.62] *	P = 0.11; I² = 60%†		
**CD8**	**Entire (9)**	0.08 [-2.31, 2.47]	P < 0.00001; I² = 94%†		***p= 0.956***
	**CT (8):**	0.60 [-2.07, 3.28]	P < 0.00001; I² = 94%†	***p= 0.506***	***p=0.728***
	GP (2)	-0.82 [-2.14, 0.50]	P = 0.77; I² = 0%		
	PE (2)	8.23 [6.28, 10.18] *	P = 1.00; I² = 0%		
	CAFI (2)	0.02 [-4.48, 4.52]	P = 0.08; I² = 67%†		
**CD3/CD8**	**Entire (3)**	0.50 [0.44, 0.56] *	p= 0.67; I² = 0%		***p= 0.091***
**CD4/CD8**	**Entire (12)**	0.30 [0.16, 0.45] *	P < 0.00001; I² = 97%†		***p= 0.229***
	**CT (11):**	0.34 [0.19, 0.49] *	P < 0.00001; I² = 97%†	***p= 0.475***	***p= 0.446***
	FOLFOX (3)	0.65 [0.56, 0.73] *	P = 0.27; I² = 23%		***p= 0.489***
	GP (2)	0.62 [0.40, 0.84] *	P = 1.00; I² = 0%		
	PE (2)	0.00 [-0.10, 0.10]	P = 1.00; I² = 0%		
	CAFI(2)	0.29 [0.10, 0.49] *	P = 0.0004; I² = 92%†		
**NK**	**Entire (2)**	5.77 [4.47, 7.08] *	P = 0.63; I² = 0%		
**ICAM-1**	**Entire (3)**	-122.91 [-137.07, -108.74] *	P = 0.67; I² = 0%		***p= 0.287***
	**CT (3):**			***p= 0.549***	
	XELOX (2)	-111.38 [-141.30, -81.46] *	P = 0.78; I² = 0%		
**IL-2**	**Entire (2)**	-4.36 [-17.45, 8.74]	(P < 0.00001; I² = 100%†		
**IL-6**	**Entire (2)**	-0.20 [-0.22, -0.19] *	P = 0.20; I² = 39%		
**TGF-β**	**Entire (2)**	-14.05 [-25.77, -2.33] *	P = 0.0001; I² = 93%†		
**TNF-α**	**Entire (2)**	-0.84 [-4.10, 2.43]	P < 0.00001; I² = 99%†		
**VEGF.a**	**Entire (8)**	-2.07 [-3.10, -1.04] *	P < 0.00001; I² = 97%†		p=0.003*
	**CT (5)**	-1.54 [-2.54, -0.54] *	P < 0.00001; I² = 96%†	***p= 0.196***	p= 0.014*
	XELOX (2)	-0.80 [-1.14, -0.46] *	P = 1.00; I² = 0%		
	**Sor (2)**	-3.75 [-8.23, 0.73]	P < 0.00001; I² = 99%†		
**AFP.a**	**Entire (5)**	-4.16 [-5.59, -2.74] *	P < 0.00001; I² = 96%†		p= 0.009 *
	**CT (4)**	-4.65 [-6.50, -2.80] *	P < 0.00001; I² = 96%†	***P=0.81***	p=0.046*
	FOLFOX (2)	-6.00 [-11.34, -0.65]	P < 0.00001; I² = 98%†		

**Note**: “. a” indicates that the result is SMD; */†: Statistically significant. ICAM-1: Intercellular Adhesion Molecule 1; IL: Interleukin; TNF-α: tumor necrosis factor alpha; TGF-β: transforming growth factor-β; VEGF: vascular endothelial growth factor; AFP: alpha fetoprotein; FMEA: fluorouracil + doxorubicin + semustine; CAFI: cisplatin + fluorouracil + azithromycin + interferon; FAP: fluorouracil + doxorubicin + cisplatin; FOLFOX: oxaliplatin + leucovorin + fluorouracil; XELOX: capecitabine + oxaliplatin; PE: pharmorubicin + cisplatin; GP: gemcitabine + cisplatin; CT: systematic chemotherapy.
