# Supplementary material for: Immunomodulatory Effect of Traditional Chinese Medicine Combined with Systemic Therapy on Patients with Liver Cancer: A Systemic Review and Network Meta-analysis
Source: J Cancer. 2022 Sep 6;13(11):3280–96. doi: 10.7150/jca.74829 (PMC9475362; doi:10.7150/jca.74829)
Supplement: Supplementary file 1 — Supplementary materials. [file jcav13p3280s1.zip › supplementary documents/Supplementary Material Document 2 .docx]

**Pubmed：**

**(((("Neoplasms, Hepatic"[Title/Abstract] OR "Neoplasms, Liver"[Title/Abstract] OR "Liver Neoplasm"[Title/Abstract] OR "Neoplasm, Liver"[Title/Abstract] OR "Hepatic Neoplasms"[Title/Abstract] OR "Hepatic Neoplasm"[Title/Abstract] OR "Neoplasm, Hepatic"[Title/Abstract] OR "Cancer of Liver"[Title/Abstract] OR "Hepatocellular Cancer"[Title/Abstract] OR "Cancers, Hepatocellular"[Title/Abstract] OR "Hepatocellular Cancers"[Title/Abstract] OR "Hepatic Cancer"[Title/Abstract] OR "Cancer, Hepatic"[Title/Abstract] OR "Cancers, Hepatic"[Title/Abstract] OR "Hepatic Cancers"[Title/Abstract] OR "Liver Cancer"[Title/Abstract] OR "Cancers, Liver"[Title/Abstract] OR "Cancer, Liver"[Title/Abstract] OR "Liver Cancers"[Title/Abstract] OR "Cancer of the Liver"[Title/Abstract] OR "Cancer, Hepatocellular"[Title/Abstract]) OR ("Liver Neoplasms"[Mesh])) AND (((((((("Drug Therapy"[Mesh]) OR ("Therapy, Drug"[Title/Abstract] OR "Drug Therapies"[Title/Abstract] OR "Therapies, Drug"[Title/Abstract] OR "Chemotherapy"[Title/Abstract] OR "Chemotherapies"[Title/Abstract] OR "Pharmacotherapy"[Title/Abstract] OR "Pharmacotherapies"[Title/Abstract])) OR ("Antineoplastic Agents"[Mesh])) OR ("****Protein Kinase Inhibitors"[Mesh])) OR ("Immunotherapy"[Mesh])) OR ("Agents, Antineoplastic"[Title/Abstract] OR "Antineoplastic Agent"[Title/Abstract] OR "Agent, Antineoplastic"[Title/Abstract] OR "Anticancer Agent"[Title/Abstract] OR "Agent, Anticancer"[Title/Abstract] OR "Anticancer Agents"[Title/Abstract] OR "Agents, Anticancer"[Title/Abstract] OR "Antineoplastic Drugs"[Title/Abstract] OR "Drugs, Antineoplastic"[Title/Abstract] OR "Antineoplastic Drug"[Title/Abstract] OR "Drug, Antineoplastic"[Title/Abstract] OR "Antineoplastic"[Title/Abstract] OR "Antitumor Drug"[Title/Abstract] OR "Drug, Antitumor"[Title/Abstract] OR "Cancer Chemotherapy Agent"[Title/Abstract] OR "Agent, Cancer Chemotherapy"[Title/Abstract] OR "Antineoplastics"[Title/Abstract] OR "Agents, Antitumor"[Title/Abstract] OR "Antitumor Agents"[Title/Abstract] OR "Drugs, Antitumor"[Title/Abstract] OR "Antitumor Drugs"[Title/Abstract] OR "Cancer Chemotherapy Agents"[Title/Abstract] OR "Cancer Chemotherapy Drugs"[Title/Abstract] OR "Chemotherapy Drugs, Cancer"[Title/Abstract] OR "Drugs, Cancer Chemotherapy"[Title/Abstract] OR "Chemotherapeutic Anticancer Agents"[Title/Abstract] OR "Chemotherapeutic Anticancer Drug"[Title/Abstract] OR "Cancer Chemotherapy Drug"[Title/Abstract] OR "Drug, Cancer Chemotherapy"[Title/Abstract] OR "Antitumor Agent"[Title/Abstract] OR "Agent, Antitumor"[Title/Abstract])) OR ("Inhibitors, Protein Kinase"[Title/Abstract] OR "Kinase Inhibitors, Protein"[Title/Abstract] OR "Inhibitor, Protein Kinase"[Title/Abstract] OR "Protein Kinase Inhibitor"[Title/Abstract] OR "Kinase Inhibitor, Protein"[Title/Abstract] OR "TKI"[Title/Abstract] OR "kinase inhibitor"[Title/Abstract])) OR ((first-line[Title/Abstract]) OR (second-line[Title/Abstract])))) AND (("Medicine, Chinese Traditional"[Mesh]) OR ("Traditional Medicine, Chinese" OR "Zhong Yi Xue" OR "Chinese Traditional Medicine" OR "Chinese Medicine, Traditional" OR "Traditional Tongue Diagnosis" OR "Traditional Chinese Medicine" OR "Chinese patent medicine" OR "traditional Chinese medicine injection " OR "Elemene injection" OR "Huachansu" OR "Cinobufacini" OR "Kanglaite" OR "Kangai injection" OR "Jinlong capsule" OR "Aidi" OR "Yadanziyouru" OR "Javanica oil emulsion" OR "Compound matrine" OR "Compound Kushen" OR "Fufangkushen" OR "yanshu" OR "Delisheng" OR "Chansu" OR "Toad venom" OR "Wutou" OR "Xiaoaiping" OR "Marsdenia Tenacissima" OR "Zhulingduotang" OR "Polyporus" OR "Astragalus polysaccharides" OR "Shenqifuzheng" OR "Shenfu" OR "Huangqi" OR "Astragalus" OR "Lentinan" OR " Shenmai" OR "Sodium Cantharidinate"))) AND ((((((((randomized controlled trial[Publication Type]) OR (controlled clinical trial[Publication Type])) OR (randomized[Title/Abstract])) OR (placebo[Title/Abstract])) OR (randomly[Title/Abstract])) OR (trial[Title/Abstract])) OR (groups[Title/Abstract])) OR (phase[Title/Abstract]))**
